# Rapidly expanding gender-affirming care based on consensus instead of evidence justifies rigorous governance and transparency

**DOI:** 10.1177/10398562241249579

**Published:** 2024-04-30

**Authors:** Andrew James Amos

**Affiliations:** Division of Tropical Health and Medicine, College of Medicine and Dentistry, 104397 James Cook University, Townsville, Australia

**Keywords:** gender dysphoria, administrative psychiatry, evidence-based medicine

## Abstract

**Objective:**

Public services offering gender-affirming care to minors have rapidly expanded across Australia. Despite limited evidence of safety and efficacy, no public information about these services is routinely available. Data from freedom of information requests sent to Australian public gender services for minors is summarised. Gender service numbers increased rapidly in Queensland (2017:190 – 2022:922) and in Victoria (2019:472 – 2023:1290). Limited transparency prevented strong confidence in the number of patients receiving hormone therapy. Staff FTE employed by gender services jumped after 2020 in NSW (to 16.7 across two sites in 2023), Queensland (to 11.4 in 2023), Victoria (to 9.4 in 2022), and WA (to 10.2 in 2023).

**Conclusions:**

Despite low confidence in their safety and efficacy, the number of patients seen by public gender services has expanded rapidly since 2018. Limited transparency makes it difficult to judge the number of patients seen, treatments provided, and outcomes achieved. Safe, effective care of this vulnerable group requires clear treatment goals, and annual reporting.

The central principle of the gender-affirming model of care (GAMOC) is that all health clinicians have an ethical responsibility not to question or evaluate patient reported gender identity, even when that identity is unstable, changes rapidly, and is co-morbid with severe mental illness.^1,2^ This research reviews methodological limitations of the rationale for GAMOC in children and adolescents that suggest the need for caution and transparency in its roll-out, reports data suggesting its roll-out in Australia has not been cautious or transparent, and discusses implications for clinical governance.

Most Australian gender services for children and adolescents are modelled on the clinical guidelines endorsed by the Australian Professional Association for Trans Health (AusPATH),^2^ in turn modelled on the World PATH (WPATH) endorsed standards of care.^1^ Absent any single description of a GAMOC and the governance framework within which it operates, it is useful to consider the AusPATH guidelines for their description of the model of care at the Royal Children’s Hospital in Melbourne, alongside the Framework for the Specialist Trans and Gender Diverse Health Service for People Under 25 Years published by NSW Health.^3^

Both documents are notable for their failure to describe any process of clinical reasoning that indicates evidence-based treatments for diagnosed illness to achieve specific benefits. While they acknowledge the existence of the mental health diagnosis ‘gender dysphoria’ (DSM-5), and the sexual health category ‘gender incongruence’ (ICD-11), they deny that either is necessary to justify social, medical, or surgical interventions to affirm a chosen gender. They note that some health systems require a medical diagnosis to access care but argue this is not medically justified. Rather, they assume access to gender-affirming interventions is a human right that should be available to anyone competent to request them. For similar reasons, they do not describe any systematic way to evaluate whether gender-affirming interventions have benefited or harmed patients but assume that access to the interventions is good of itself as the satisfaction of a human right.^1,2^

Both documents explicitly acknowledge their recommendations are based on clinician consensus because of the lack of reliable evidence about the benefits and harms of gender-affirming interventions.^1,2^ However, they don’t acknowledge that ‘gender dysphoria’ and ‘gender incongruence’ are also based on clinician consensus because of the lack of reliable evidence.^4,5^ By contrast, the reliability of DSM-5 diagnoses like Schizophrenia were demonstrated by field trials involving hundreds of clinicians and thousands of patients.^6^

In the context of the lack of empirical evidence for a gender diversity nosology, it is important that the diagnostic evolution of gender diversity from transsexualism to gender dysphoria and gender incongruence has been influenced by organisations with the explicit goal of ‘depathologizing’ gender diversity.^7–9^ Neither DSM-5 nor ICD-11 empirically justified these changes, while both acknowledged the influence of non-clinician stakeholders.

Given the methodological limitations and non-clinician influence on the development and provision of the GAMOC,^7,8^ it is concerning that clinician whistleblowers and legal actions by patients and families claiming harm led to the Cass Review of one of the most prestigious centres offering the GAMOC in the UK. The interim report concluded the GAMOC at the Tavistock Institute was not evidence-based, failed to collect data on the outcomes of its treatment, encouraged clinicians to affirm gender identity even when this conflicted with high-quality care, and often failed to assess mental health.^10^

A major gap in the Australian framework for the GAMOC is the failure to describe how to detect, encourage, and support those patients who detransition. The WPATH standards recommend a multidisciplinary approach, but don’t describe how to implement this (pS41).^1^ Neither the AusPATH guidelines nor the NSW Health Framework even contain the word ‘detransition’.^2,3^

In the context of these methodological limitations, alongside the reluctance of GAMOC advocates to publicly discuss their methods and results,^8,11^ and the existence of stakeholder groups with a demonstrable record of influence on the medical framework for the management of gender diversity,^7^ the roll-out of the GAMOC in Australia demands rigorous clinical governance and external transparency. Box 1 describes the limited information available from Freedom of Information requests about the rapid expansion of Australian public gender services for minors as a starting point for the Discussion.

**Box 1 – the growth of gender services in Australia revealed by freedom of information requests**

There are no publicly available descriptions of the number, composition, or treatment of patients seen by Australian gender services. As far as I have been able to determine, none of these services currently or historically publish any details of their clinical operations. The only source of information about their operations appears to be via Freedom of Information requests, which can be made by any Australian citizen.

By his own report, starting in 2017, a NSW MP, Greg Donnelly, made annual requests to all gender services operating in Australian public Hospital and Health Services (HHS) using a standard set of questions (Table 1). For each service, the first set of questions included a request for the same set of information back to 2014, or the first year of operation of the gender service if later than 2014.

**Table 1.** Freedom of information questions.^a^

1) As at 24^th^ September 20XX, how many children and adolescents, who are transgender or gender diverse are receiving treatment at the HHS gender service? Can the figures reflect as follows:
a) The number of those whose natal sex was female?
b) The number of those whose natal sex was male?
c) The number of those who were born intersex?
2) As at 24^th^ September 20XX, how many children and adolescents, who are transgender or gender diverse are receiving stage one puberty blocker treatment at the HHS Gender Service? Can the figures reflect as follows:
a) The number of those whose natal sex was female?
b) The number of those whose natal sex was male?
c) The number of those who were born intersex?
3) As at 24th September 20XX, how many children and adolescents, who are transgender or gender diverse are receiving stage two gender-affirming hormone treatments at the HHS Gender Service? Can the figures reflect as follows:
a) The number of those whose natal sex was female?
b) The number of those whose natal sex was male?
c) The number of those who were born intersex?
4) As at 24^th^ September 20XX, how many employees including consultants (measured as full-time equivalents (FTE’s)) worked at the HHS Gender Service?

^a^ – 24^th^ September 20XX and HHS Gender Service are used as placeholders – individual requests used different dates/years and the name of the specific service, for example, ‘The Sydney Children’s Hospitals Network Gender Clinic’.

The questions sent to each service requested a minimum set of data necessary to understand the overall number of patients being treated at a point in time of each year, the type of treatment offered (across the three basic levels of assessment, treatment with puberty blockers, and treatment with gender-affirming hormones), patients’ biological sex, and the workforce manning the gender service.

The NSW MP Greg Donnelly made the data obtained via these FOI requests freely available to the author without any conditions. No payment in money or in kind was made for the data, and the author has no other connection with Mr Donnelly. The author became aware of the existence of the data after making a presentation about the political implications of the GAMOC at the NSW Parliament on 6^th^ February, 2024. While supplying the data, Mr Donnelly stated that once FOI requests have been answered they become part of the public domain, a fact confirmed by the FOI websites of each jurisdiction listed in Table 2.

**Table 2.** Australian gender service freedom of information requests.

**Freedom of information framework in Australia**	
*Right to information as a principle of good governance in Australian Jurisdictions*	
Transparency is a principle of good governance enshrined in the legislation of all Federal, State, and Territory governments in Australia. As described by the office of the Australian information commissioner of the Australian Government, all Australian citizens have the ‘right to request access to government-held information. This includes information they hold about you or about government policies and decisions’. It describes organisations that are generally exempt from FoI requests, usually on national security grounds, and the conditions necessary for a FoI request to non-exempt organisations to be rejected (such as non-existent information, personal information about individuals, among others)^12^	
*Practical guide to accessing government information through FoI requests*	
The best single practical resource for understanding and leveraging Freedom of Information requests in Australia is the *Right to Know* organisation which provides a single access point for making FoI requests of government organisations, catalogues a list of public organisations which are subject to FoI requests, gives advice on how to make a successful request to each of those organisations, and makes publicly available many responses to past FoI requests to each of those organisations.^13^ https://www.righttoknow.org.au/	
*FoI Requests by Jurisdiction*	
FoI requests of Australian government bodies must be addressed to specific offices of specific organisations, and must request specific documents, rather than requesting particular information. The data for the current research was obtained by FoI requests to specific offices responsible for responding to FoI requests, identifying specific agencies holding the required information, under the provisions of specific legislation and returning specific documents, all of which are summarised below alongside standard costs as of 2024. The information provided in response to successful FoI requests becomes part of the public domain but there is no centralised system for accessing them. The *Right to Know* website above has a partial list, and it is possible to request previous FoI responses from the original responding office using the references provided; processing charges should be minimal/free for existing documents	
NSW	*Office responsible for FoI requests*: NSW Health, right to information team – https://www.health.nsw.gov.au/gipaa/Pages/default.aspx
	*Agency*: Sydney Children’s Hospital Network
	*Legislation*: Government Information (Public Access) Act 2009 s9(3)
	*References*: SCHN18/7854, SCHN19/8491, SCHN20/9567, SCHN22/2274, SCHN22/10947, SCHN23/12795
	*Cost for FoI request as at 11.03.24*: $0 – Agencies encouraged to provide FoI responses free of charge; in some circumstances they can charge $30 per hour spent collecting information; these responses were provided free of charge
NSW	*Office responsible for FoI requests*: NSW Health, Right to Information team – https://www.health.nsw.gov.au/gipaa/Pages/default.aspx
	*Agency*: Maple Leaf House
	*Legislation*: Government Information (Public Access) Act 2009 s9(3)
	*References*: GIPA22_036, GIPA23-030
	*Cost for FoI request as at 11.03.24*: Agencies encouraged to provide FoI responses free of charge; in some circumstances, they can charge $30 per hour spent collecting information; the 2023 response cost $230 in total
QLD	*Office responsible for FoI requests*: Queensland Health – Health Information Services - https://www.health.qld.gov.au/system-governance/contact-us/access-info/rti-application
	*Agency*: Queensland Children’s Gender Service
	*Legislation*: Right to information Act 2009 s30(2)
	*References*: 18/101, 19/127, 20/118, 21/335, 22/204, 23/250
	*Cost for FoI request as at 11.03.24*: Application fee of $55.75, with variable processing charges – the 2023 request had no processing charges due to processing of application in less than 5 hours
SA	*Office responsible for FoI requests*: SA Health – Freedom of Information Office – https://www.sahealth.sa.gov.au/wps/wcm/connect/public+content/sa+health+internet/about+us/accessing+information/freedom+of+information
	*Agency*: Women’s and Children’s Health Network, Women’s and Children’s Hospital
	*Legislation*: Freedom of Information Act, 1991
	*References*: WCHN 181586, WCHN 193198, FOI-2022 – 00089
	*Cost for FoI request as at 11.03.24*: $40.75 application fee plus processing charges if applicable (not charged for these responses)
TAS	*Office responsible for FoI requests*: Tasmanian Department of Health – Legal Services – https://www.health.tas.gov.au/about/routine-disclosures/right-information
	*Agency*: Royal Hobart Hospital Tasmanian Gender Service
	*Legislation*: Right to Information Act 2009 s3
	*References*: RTI202223-173
	*Cost for FoI request as at 11.03.24*: $44.50 application fee which may be waived in some cases, no routine processing fee
VIC	*Office responsible for FoI requests*: Royal Children’s Hospital – Freedom of Information Office – https://www.rch.org.au/foi/
	*Agency*: Royal Children’s Hospital Melbourne Gender Service
	*Legislation*: Freedom of Information Act 1982
	*References*: FOI20180642, FOI20190624, FOI20200549, FOI20220152, FOI20220727, FOI20230812
	*Cost for FoI request as at 11.03.24*: $31.80 application fee plus variable processing costs (no variable costs for these applications)
WA	*Office responsible for FoI Requests*: WA Department of Health – Freedom of Information team – https://www.health.wa.gov.au/About-us/Freedom-of-Information
	*Agency*: WA Child and Adolescent Health Service – Gender Diversity Service
	*Legislation*: Freedom of Information Act 1992
	*References*: CF-23-8169, 19/20687, 20/21558, CF-22-1886, CF-23-57
	*Cost for FOI request as at 11.03.24*: $30 application fee; potential processing charges for work-intensive requests (none for these requests)

Table 2 ^12,13^ describes the framework for FoI requests in Australian federal and state/territory jurisdictions under which these requests were made, including the legislation relied upon, the agencies queried, and the costs incurred. All costs for this research were paid for by the office of the NSW MP who made them. Successful FoI requests become part of the public domain (with limited exceptions which do not apply to these documents), and these responses were provided to the researcher without charge.

This data was collated and presented in graphical form, and tabulated to summarise which gender services exist, when they started, and what information they were willing to provide based on a standard set of questions under FOI laws.

The summary of the framework for FoI requests provided in Table 2 includes links to the FoI services provided by each gender service or its governing body. All Australia’s jurisdictions positively endorse the right of citizens to access government held non-personal information with limited exceptions largely based on anticipated harms, such as national security information.

Table 3 summarises known gender services provided by Australian public health services including services offered, primary website, and a brief description of the service and its target population.

**Table 3.** Public Australian Health Services offering Gender-Affirming Care to minors.

Service	Description
NSW	
Sydney Children’s Hospitals Network	https://www.health.nsw.gov.au/lgbtiq-health/Pages/tgd-health-service.aspx
	Sydney/metropolitan-based provision of gender-affirming care is moving from a single site service at The Children’s Hospital Westmead (for children/adolescents older than 9 and younger than 18) to a hub model starting in 2024 in which The Children’s Hospital Westmead will offer services to children <16yo and South Eastern Sydney Local Health District will offer services to adolescents >15yo. The model of care is based on the AusPATH guidelines and WPATH standards of care
	**Services offered: Assessment, puberty blockers, (hormone therapy to start from 2024)**
Maple Leaf House	https://www.hnekidshealth.nsw.gov.au/specialist_services/gender
	NSW rural and regional gender-affirming care was established in 2021 at Maple Leaf House by Hunter New England Local Health District, based on the AusPATH guidelines and WPATH standards of care
	**Services offered: Assessment, puberty blockers, hormone therapy**
Victoria	
Monash Health Gender Clinic	https://www.monash.health.org/services/gender-clinic/
	Statewide service for gender diverse people 16yo and older consistent with the AusPATH and WPATH model of care. Not included in FOI requests
	**Services offered: Assessment/referral.** (Notes partnerships with services offering puberty blockers, hormone therapy, and surgeries)
Royal Children’s Hospital Gender Service	https://www.rch.org.au/adolescent-medicine/gender-service/
	Statewide service for gender diverse people up to 16yo; developed the AusPATH guidelines and consistent with the WPATH standards
	**Services offered: Assessment, puberty blockers, hormone therapy**
Qld	
Queensland Children’s Hospital Gender Service	https://www.childrens.health.qld.gov.au/services/gender-service/gender-service-queensland-childrens-hospital
	Statewide service for gender diverse people under 17yo consistent with the AusPATH and WPATH model of care
	**Services offered: Assessment, puberty blockers, hormone therapy**
WA	
Perth Children’s Hospital Gender Diversity Service	https://pch.health.wa.gov.au/Our-services/Mental-Health/Gender-Diversity-Service
	Statewide service for gender diverse patients <18yo. Does not mention the model of care
	**Services offered: Assessment, puberty blockers, hormone therapy**
SA	
Women’s and Children’s Hospital Gender Diversity team	https://www.wch.sa.gov.au/patients-visitors/family-support-services/gender-diversity
	Statewide service for gender diverse patients <18yo consistent with the AusPATH clinical guidelines
	**Services offered: Assessment, puberty blockers, hormone therapy**
Tasmania	
Tasmanian Gender Service	https://www.health.tas.gov.au/health-topics/sexual-and-reproductive-health/tasmanian-gender-service-tgs
	Statewide service for gender diverse patients <18yo consistent with the AusPATH clinical guidelines. Notes a close relationship with the RCH Gender Service in Melbourne
	**Services offered: Assessment, puberty blockers, hormone therapy**
NT	
No public gender service identified	https://nt.gov.au/wellbeing/transgender-and-gender-diverse-services
ACT	
New paediatric gender service announced in June 2023:	https://nt.gov.au/wellbeing/transgender-and-gender-diverse-services

There was significant variation in the information provided by different gender services in response to the FOI requests (Table 4). Maple Leaf House (MLH) in NSW and the Queensland Children’s Hospital (QCH) did not supply information about patients’ natal sex, and MLH did not provide the number of patients receiving hormone therapy, on the basis that this information was not available from their health databases. It was not clear from their responses why the RCH in Victoria and the QCH service reported the number of new patient treatments each year rather than the number of patients receiving each type of treatment.

**Table 4.** Transparency of public Australian gender services for minors including responses to freedom of information requests.

Service	Model of care	Client number	Services provided	Natal sex	Workforce
NSW – SHCN	AusPATH/WPATH	Yes	Yes	Yes	Yes
NSW – Maple Leaf	AusPATH/WPATH	Yes	**Partial** ^a^	**No** ^a^	Yes
Victoria – RCH	AusPATH/WPATH	**Partial** ^b^	**Partial** ^b^	Yes	Yes
Qld – QCH	AusPATH/WPATH	**Partial** ^b^	**Partial** ^b^	**No** ^**c**^	Yes
WA – PCH	Not specified	Yes	Yes	Yes	Yes
SA – WCH	Not specified	Yes	Yes	Yes	**No** ^d^
Tasmania – DoH^e^	AusPATH	**No**	**No**	**No**	Yes

^a^Maple Leaf House did not provide either patients’ natal sex or the number of patients receiving hormone therapy with the rationale that the database ‘does not hold the information in the format requested’.

^b^Both Victorian and Queensland services (after 2019) provided annual initiation of medical treatments rather than number of ongoing clients receiving treatments.

^c^Queensland Health did not describe reasons that information on natal sex was not supplied.

^d^SA Health reported that it was not possible to identify the Gender Service workforce on the HHS database.

^e^FOI requests refused by Tasmania Health due to small number of patients judged to increase risk of violating anonymity.

For each service, Figure 1 charts the total number of patients, patients receiving puberty blockers/hormone therapy, annual initiation of puberty blockers/hormone therapies per year, and total FTE of clinical staff working for the gender service. Figure 1 demonstrates that the increase in total FTE in NSW was due to the creation of a new gender service in 2022 (Maple Leaf House) while the established service (SCHN) had essentially plateaued by 2020.

## Discussion

**Figure 1.**
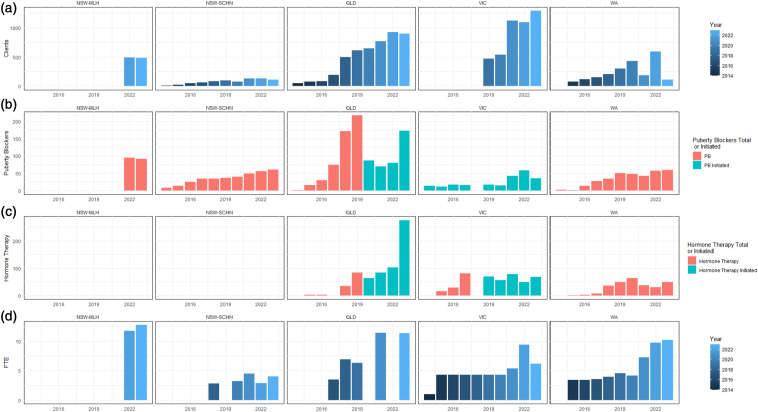
(a) Total clients per gender service each year (point in time); (b) Patients receiving puberty blockers per gender service each year (total at point in time or initiated per year); (c) Patients receiving hormone therapy per gender service each year (total at point in time or initiated per year)*; (d) Full-time equivalent staff per gender service each year. *Note: Although their website suggests they do offer hormone therapy, the FOI response by the NSW-MLH did not include any data about the number of patients receiving it; the NSW-SCHN website indicates that they do not offer hormone therapy.

The GAMOC endorsed by AusPATH and WPATH appears to dominate the management of gender diverse minors in Australia’s public health services despite the lack of evidence about its benefits, harms, and diagnostic reliability. While there is no routine and public information about the nature of treatment, number of patients treated, or outcomes of treatments provided by these services, FOI requests confirm that there has been a rapid expansion in service availability, staff numbers, and patients since the release of the AusPATH endorsed guidelines in 2018^14^ (Figure 1).

In the absence of the sort of standardised annual reports provided by most other parts of the public health service, it is impossible to draw strong conclusions about the strengths and limitations of the services provided to gender diverse patients in Australia. The most remarkable patterns revealed by FOI data are the rapid expansion of patients, staff, and interventions in Queensland since 2018, the rapid expansion of patients but not staff or interventions in Victoria, and the relatively restrained increase in patients and interventions but rapid increase in staff in NSW.

Perhaps the greatest anomaly among these data is the complete absence of any information about hormone therapies delivered by public health services in NSW. This is particularly evident for Maple Leaf House, which covers rural/regional NSW and yet in its 2 years of operation has jumped ahead of the metropolitan Sydney service in staffing (12.76 v 4 FTE in 2023), ongoing clients (482 v 108), and in the provision of puberty blockers (91 v 61).

### The need for strong and transparent clinical governance of gender-affirming care

The lack of a high-quality evidence base for the diagnosis of gender dysphoria and the interventions recommended by the GAMOC suggests this model requires a higher than usual standard of clinical governance and transparency. However, as demonstrated by this research, even with the aid of FOI requests, it is impossible to draw any strong conclusions about the nature, quality, or outcomes of the GAMOC in Australia, other than its rapid expansion in the absence of transparent oversight.

Some gender-affirming advocates have argued that the usual level of scientific scrutiny required to establish novel medical paradigms, such as the use of randomised clinical trials to confirm the efficacy, effectiveness, and lack of harm of particular treatments, is unethical.^15^ Others have suggested that the application of evidence-based medicine to the treatment of gender diversity constitutes pathologisation which is itself directly harmful to gender diverse people.^16^ The RANZCP’s original endorsement of the GAMOC was consistent with these arguments, which prevent the only means of confirming whether the GAMOC helps or harms patients. This appears to have been recognised in the RANZCP’s reversal of that endorsement and the promulgation of a position statement that explicitly acknowledges there is no strong evidence regarding the outcomes of the GAMOC.^17,18^

The dangers of expanding a novel treatment paradigm in the absence of evidence are beginning to become apparent with respect to the GAMOC. One of the most powerful emotional arguments that has been used in support of gender affirmation is that it reduces the risk of suicide in gender diverse patients.^16^ However, research into suicide in gender diverse patients has involved multiple methodological limitations, including a focus on measures other than completed suicide, a lack of appropriate controls, and a failure to consider the differential influence of gender diversity and co-morbid psychiatric illness and symptoms. A new nationwide Finnish register study has found that while there was a higher rate of completed suicide in gender diverse patients under 23yo (0.3% vs 0.1% in controls), gender dysphoria was not predictive of suicide after controlling for psychiatric treatment history.^19^

The Cass Review reported that the GAMOC provided at the Tavistock was associated with diagnostic overshadowing, where the diagnosis of gender dysphoria led to neglect of other health and mental health conditions.^10^ If it is true that gender-affirming care reduces the probability of the diagnosis and treatment of psychiatric illness, and the risk of suicide in gender dysphoric patients is associated with psychiatric illness and not with gender dysphoria, then paradoxically it is plausible that treatment under the GAMOC would increase the risk of suicide, rather than decrease this risk.

While this line of reasoning is speculative, it illustrates that there are plausible risks associated with the GAMOC provided at Australia’s gender services which can only be confirmed or denied by high-quality research. The fact that Australia’s gender services have expanded so rapidly in the absence of such research suggests that it is not a priority of those services. This in turn reinforces the need for greater than usual transparency in demonstrating that rigorous clinical governance has been practiced in the oversight of these services.

## Conclusions

The GAMOC has expanded rapidly across Australia despite the lack of evidence for the diagnosis of gender dysphoria or the interventions it recommends. While advocates have argued that external scrutiny may be harmful to gender diverse patients, adopting this approach would have prevented the demonstration that gender diverse patients are not at higher risk of completed suicide than controls when psychiatric co-morbidities are taken into account. The uncertain foundations of the GAMOC and the vulnerability of gender diverse patients demand a high level of external scrutiny. At a minimum, public gender services including those discussed here should routinely publicly report the number of new referrals and ongoing patients, relevant clinical characteristics such as natal sex and gender identity, and number of newly initiated and ongoing treatments by type. In addition, gender services should be required to define how they measure successful treatment and common harms such as side-effects and detransition and report aggregate patient outcomes. The clinical governance practice of more established medical, surgical, and psychiatry units in tracking indicators such as length of stay, readmission, surgical complications, and objectively measured functional or symptomatic improvements might provide a useful starting point for gender services.

Finally, given the history of non-clinician influence over this area of medicine,^7,8,10^ those with responsibility for clinical governance should be aware of the possibility of conscious or unconscious bias in the implementation of the GAMOC by gender services. Given the changing international context and potential legal liability revealed by the Cass Review,^10^ it would be prudent for health authorities, including public HHSs with gender services, to acknowledge the uncertainties of the GAMOC, seek the opinion of critical voices, and publicly address criticisms. Correcting the failure of the GAMOC to manage or even monitor the rate of detransition of patients seen by gender services would be an ideal indicator of progress towards the goal of high-quality clinical governance of the GAMOC in Australia’s public HHSs.
